# Non-Alcoholic Steatohepatitis (NASH) and Liver Fibrosis: Molecular and Multicellular Control of Evolving Diseased States

**DOI:** 10.3390/cells11162551

**Published:** 2022-08-17

**Authors:** Jérôme Eeckhoute

**Affiliations:** U1011-EGID, Institut Pasteur de Lille, CHU Lille, Inserm, Univ. Lille, F-59000 Lille, France; jerome.eeckhoute@inserm.fr

Non-alcoholic fatty liver disease (NAFLD), the most common chronic liver disease, has emerged as a major threat to public health [1]. Indeed, obesity and type 2 diabetes are independent risk factors of NAFLD for which increases contribute to the devasting consequences of the metabolic disease pandemic [2]. Importantly, NAFLD presents with a range of conditions initiated by relatively benign hepatic steatosis, which can evolve to non-alcoholic steatohepatitis (NASH); fibrosis; and ultimately, cirrhosis [3]. Advanced stages promote hepatocellular carcinoma and/or liver failure [1]. In this context, NASH has been the fastest growing indication for liver transplantation in Western countries and is expected to become the leading cause of liver transplant in the United States [4,5].

NASH is characterized by an escalation in the disease complexity, where suffering hepatocytes trigger inflammation and, secondarily, liver fibrosis [1,2,3]. These processes are increasingly recognized as being driven by dynamic and coordinated changes in the complexity and activities of the various liver cell populations [6]. Indeed, in addition to hepatocytes and immune cells, resident or recruited, the role exerted by cholangiocytes, (myo)fibroblasts, and endothelial cells is also now being analyzed in detail (e.g., [7,8,9]). In this context, our understanding of the molecular and multicellular mechanisms underlying the various NAFLD stages is still limited and defining how/why the disease evolves to more advanced stages has remained challenging. For instance, the kinetic, spatial organization, and functional hierarchy of changes in cellular states and activities largely remain to be described together with the identity and integration of intercellular signals involved. At the individual cell-type level, molecular (mal)adaptations accompanying the disease progression, including transcriptomic dysregulation, also need to be further characterized. Indeed, diagnosis and therapeutic treatment of NASH and fibrosis remain controversial and of limited efficacy [10,11,12] and would greatly benefit from a greater understanding of the molecular mechanisms at play in the multicellular processes defining advanced NAFLD stages. The purpose of this Special Issue is to highlight recent findings in those areas, enlightening how NASH and its associated liver fibrosis develop through coordinated modulation of the activities of different liver cell-types.

## References

[1] Loomba R., Friedman S.L., Shulman G.I. (2021). Mechanisms and disease consequences of nonalcoholic fatty liver disease. Cell.

[2] Haas J.T., Francque S., Staels B. (2016). Pathophysiology and Mechanisms of Nonalcoholic Fatty Liver Disease. Annu. Rev. Physiol..

[3] Schwabe R.F., Tabas I., Pajvani U.B. (2020). Mechanisms of Fibrosis Development in NASH. Gastroenterology.

[4] Wong R.J., Aguilar M., Cheung R., Perumpail R.B., Harrison S.A., Younossi Z.M., Ahmed A. (2015). Nonalcoholic steatohepatitis is the second leading etiology of liver disease among adults awaiting liver transplantation in the United States. Gastroenterology.

[5] Younossi Z.M., Stepanova M., Ong J., Trimble G., AlQahtani S., Younossi I., Ahmed A., Racila A., Henry L. (2021). Nonalcoholic Steatohepatitis Is the Most Rapidly Increasing Indication for Liver Transplantation in the United States. Clin. Gastroenterol. Hepatol..

[6] Wallace S.J., Tacke F., Schwabe R.F., Henderson N.C. (2022). Understanding the cellular interactome of non-alcoholic fatty liver disease. JHEP Rep..

[7] Guilliams M., Bonnardel J., Haest B., Vanderborght B., Wagner C., Remmerie A., Bujko A., Martens L., Thone T., Browaeys R. (2022). Spatial proteogenomics reveals distinct and evolutionarily conserved hepatic macrophage niches. Cell.

[8] Terkelsen M.K., Bendixen S.M., Hansen D., Scott E.A.H., Moeller A.F., Nielsen R., Mandrup S., Schlosser A., Andersen T.L., Sorensen G.L. (2020). Transcriptional dynamics of hepatic sinusoid-associated cells after liver injury. Hepatology.

[9] Haas J.T., Vonghia L., Mogilenko D.A., Verrijken A., Molendi-Coste O., Fleury S., Deprince A., Nikitin A., Woitrain E., Ducrocq-Geoffroy L. (2019). Transcriptional Network Analysis Implicates Altered Hepatic Immune Function in NASH development and resolution. Nat. Metab..

[10] Rinella M.E., Loomba R., Caldwell S.H., Kowdley K., Charlton M., Tetri B., Harrison S.A. (2014). Controversies in the Diagnosis and Management of NAFLD and NASH. Gastroenterol. Hepatol..

[11] Leslie M. (2015). The liver’s weighty problem. Science.

[12] Friedman S.L., Pinzani M. (2022). Hepatic Fibrosis 2022: Unmet Needs and a Blueprint for the Future. Hepatology.

